# Identifying Aging-Related Biomarkers and Immune Infiltration Features in Diabetic Nephropathy Using Integrative Bioinformatics Approaches and Machine-Learning Strategies

**DOI:** 10.3390/biomedicines11092454

**Published:** 2023-09-04

**Authors:** Tao Liu, Xing-Xing Zhuang, Jia-Rong Gao

**Affiliations:** 1 Department of Pharmacy, The First Affiliated Hospital of Anhui University of Chinese Medicine, Hefei 230012, China; 2020205219007@stu.ahtcm.edu.cn; 2 College of Pharmacy, Anhui University of Chinese Medicine, Hefei 230011, China; 3 Department of Pharmacy, Chaohu Hospital of Anhui Medical University, Chaohu 238000, China; zxx900913@126.com

**Keywords:** diabetic nephropathy, aging, diagnostic biomarker, immune cell infiltration, molecular docking

## Abstract

Background: Aging plays an essential role in the development of diabetic nephropathy (DN). This study aimed to identify and verify potential aging-related genes associated with DN using bioinformatics analysis. Methods: To begin with, we combined the datasets from GEO microarrays (GSE104954 and GSE30528) to find the genes that were differentially expressed (DEGs) across samples from DN and healthy patient populations. By overlapping DEGs, weighted co-expression network analysis (WGCNA), and 1357 aging-related genes (ARGs), differentially expressed ARGs (DEARGs) were discovered. We next performed functional analysis to determine DEARGs’ possible roles. Moreover, protein–protein interactions were examined using STRING. The hub DEARGs were identified using the CytoHubba, MCODE, and LASSO algorithms. We next used two validation datasets and Receiver Operating Characteristic (ROC) curves to determine the diagnostic significance of the hub DEARGs. RT-qPCR, meanwhile, was used to confirm the hub DEARGs’ expression levels in vitro. In addition, we investigated the relationships between immune cells and hub DEARGs. Next, Gene Set Enrichment Analysis (GSEA) was used to identify each biomarker’s biological role. The hub DEARGs’ subcellular location and cell subpopulations were both identified and predicted using the HPA and COMPARTMENTS databases, respectively. Finally, drug–protein interactions were predicted and validated using STITCH and AutoDock Vina. Results: A total of 57 DEARGs were identified, and functional analysis reveals that they play a major role in inflammatory processes and immunomodulation in DN. In particular, aging and the AGE-RAGE signaling pathway in diabetic complications are significantly enriched. Four hub DEARGs (CCR2, VCAM1, CSF1R, and ITGAM) were further screened using the interaction network, CytoHubba, MCODE, and LASSO algorithms. The results above were further supported by validation sets, ROC curves, and RT-qPCR. According to an evaluation of immune infiltration, DN had significantly more resting mast cells and delta gamma T cells but fewer regulatory T cells and active mast cells. Four DEARGs have statistical correlations with them as well. Further investigation revealed that four DEARGs were implicated in immune cell abnormalities and regulated a wide range of immunological and inflammatory responses. Furthermore, the drug–protein interactions included four possible therapeutic medicines that target four DEARGs, and molecular docking could make this association practical. Conclusions: This study identified four DEARGs (CCR2, VCAM1, CSF1R, and ITGAM) associated with DN, which might play a key role in the development of DN and could be potential biomarkers in DN.

## 1. Introduction

Diabetic kidney disease (DN), one of the most common microvascular effects of diabetes, is the leading cause of end-stage renal disease (ESRD) worldwide [1]. The major lesion of DN, visible in the glomeruli, is characterized by extracellular matrix deposition and basement membrane thickening [2]. There is still much to learn about the pathogenesis and etiology of DN. Historically, the presence of microalbuminuria (MA) and the progression of diabetes mellitus (DM) have been used to make an early diagnosis of DN [3]. This approach is ineffective, though, as only 30% of cases had pathology confirm them, and the remaining cases were DM with primary glomerular illnesses present [4]. The lack of complete and efficient therapy for DN makes it the main contributor to renal dysfunction and ESRD development [5]. Even though there are several potential causes of DN, including obesity, heredity, and environment, the general pathophysiological pathways remain unknown. Consequently, understanding the causes of DN is essential for discovering and identifying diagnostic biomarkers for the incidence and progression of DN.

Recent research shows that DN is highly associated with accelerated aging in various cell types, including endothelial, tubular, and podocyte cells [6,7,8]. Increased cortical surface roughness, the number of cysts, and reduced cortical volume are just a few of the macrostructural changes that the aging kidneys experience [9]. These changes correspond to the typical microstructural features of glomerulosclerosis, tubular atrophy, interstitial fibrosis, and nephron loss [10]. Notably, mesangial and tubular cells were susceptible to direct cellular senescence induction by hyperglycemia [11,12,13,14]. Low-grade inflammation and cellular aging could be promoted by excessive glucose, which is also capable of making macrophages release SASP components [15]. Along with hyperglycemia, AGE generation, oxidative stress induction, chronic persistent inflammation, glucose toxicity, and lipid metabolism problems could all work in concert to foster the development of a favorable milieu for aging cells [16]. Aging is also accompanied by dysregulation of the immune system, which is characterized as immune aging and involves impaired immune responses and overwhelming inflammation [17]. Immune dysregulation and inflammation linked with immune aging have been identified as risk factors for a wide range of age-related illnesses [18,19]. Immune aging can eventually lead to increased vulnerability to age-related comorbidities such as cancer, autoimmune illnesses, and infectious diseases [20].

In this investigation, we employed gene expression profiles specific to DN, combined with aging-related databases, bioinformatic analyses, and validation tests, to identify aging-associated genes that serve as potential biomarkers for DN development. Furthermore, we elucidated the immune mechanisms underlying DN by conducting an immune infiltration analysis, thereby uncovering the immunological basis of these biomarkers. Our study aimed to provide novel insights into the intricate relationship between aging, immune responses, and the progression of DN.

## 2. Material and Methods

**Figure 1:**
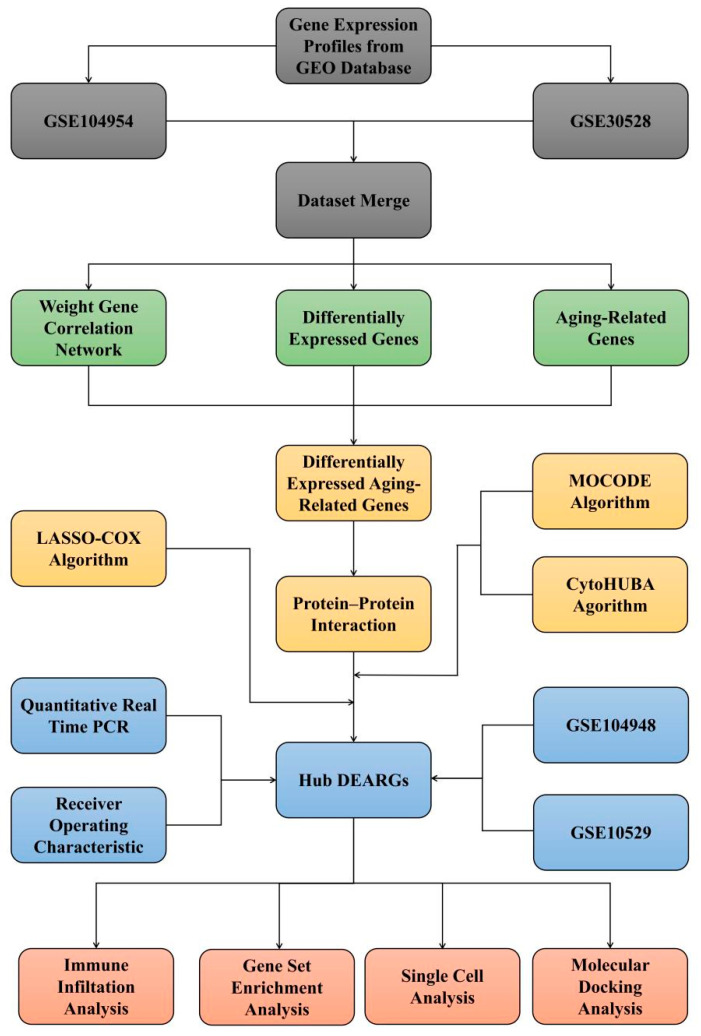
The study flowchart for DN based on integrative bioinformatics approaches and machine-learning strategies.

This study’s objective was to investigate DN-related gene sets via the lens of ARGs. The study flowchart is depicted in Figure 1.

### 2.1. Data Preprocessing and Differentially Expressed Genes (DEGs) and Aging-Related Genes (ARGs) Identifying in DN

First, to investigate the DEGs in DN patients compared to healthy individuals, we obtained the gene expression profiles of DN patients from the publicly available GEO database. The NCBI GEO GSE104954 and GSE30528 datasets provided datasets with clinical details on DN and healthy kidney samples. The 7 DN kidney tissues and 18 normal tissues in the GSE104954 data set were based on the Affymetrix Human Genome U133 Plus 2.0 Array of the GPL22945 platform. The 9 DN kidney tissues and 13 normal tissues in the GSE30528 data set were based on the Affymetrix Human Genome U133A 2.0 Array of the GPL571 platform. We processed the datasets using the inSilicoMerging [21] R program to combine the various datasets. In order to exclude group effects, we also applied the Johnson et al. technique [22]. The follow-up analysis of this study includes a total of 16 DN samples and 31 normal tissue samples. The limma R tool was then used in differential analysis to find the genes that differ between the DN group and the control group [23]. The |fold-change (FC)| > 1.5 and *p*-value < 0.05 were the statistical thresholds for screening RNA expression. Subsequently, in order to obtain ARGs, we retrieved 1357 ARGs from the GeneCards database (Supplementary Table S1) with a relevance score of greater than 5 [24].

### 2.2. Identification of Clinically Significant Modules Based on Weight Gene Correlation Network Analysis (WGCNA)

Next, we employed the WGCNA to further identify gene clusters that play crucial roles in the onset and progression of DN. In order to create the scale-free co-expression network, we removed outlier DEGs and probes using the R program WGCNA. To be more precise, all pair-wise genes were first subjected to the average linkage approach and Pearson’s correlation matrices. A soft-thresholding parameter, β, emphasized strong gene connections and penalized weak ones. In order to measure the network connectivity of a gene, which is defined as the sum of its adjacency with all other genes for network gene ratio, the adjacency was transformed into a topological overlap matrix (TOM) after selecting the power of 6, and the corresponding dissimilarity (1-TOM) was calculated. Average linkage hierarchical clustering was carried out using the TOM-based dissimilarity measure with a minimum size (gene group) of 10 for the gene dendrogram in order to arrange genes with comparable expression profiles into gene modules. We determined the dissimilarity of the module eigengenes, selected a cut line for the module dendrogram, and combined several modules in order to further investigate the module.

### 2.3. Functional Enrichment Analysis of DEGs and DEARGs

Furthermore, we performed functional enrichment analysis to explore the biological roles of DEGs and DEARGs in DN. Based on the Molecular Signatures Database (MSigDB) and the DAVID database, we obtained gene annotations for the Human Phenotype Ontology (HPO), Gene Ontology (GO), and Kyoto Encyclopedia of Genes and Genomes (KEGG), respectively. We used the ClusterProfiler R package and DAVID database to perform HPO, GO, and KEGG function analysis to acquire the results of the DEGs enrichment. Statistical significance was defined as *p*-value < 0.05. The maximum and minimum gene sets are 5000 and 5, respectively.

### 2.4. Identification of Hub Biomarkers Based on Protein–Protein Interaction (PPI) and Machine Learning Algorithm

Moreover, we employed PPI analysis and a machine learning algorithm to identify hub biomarkers that play pivotal roles among DEARGs. The STRING database was utilized to examine the interactions of different module genes with filtering criteria (score > 0.4). The network was displayed using Cytoscape 3.8.1. The Molecular Complex Detection (MCODE) plug-in for Cytoscape was used to examine the primary functional modules. These criteria for selection are defined as follows: K Core = 2, Cut Grade = 2, Maximum Depth = 100, Cut Node Score = 0.3. Each node gene is scored using the Maximum Clique Centrality (MCC) by the cytoHubba plug-in for Cytoscape. The top 10 nodal genes of each algorithm’s MCC score were used to screen for the pivot genes. Then, the hub biomarkers were found using Cox regression with the Least Absolute Shrinkage and Selection Operator (LASSO). Based on the 3-fold cross-validation method, we calculated the penalty parameter, selected the best value corresponding to the lowest cross-validation error, and listed the gene names matching that value utilizing the “glmnet” software package. The GeneMANIA database (http://genemania.org/ (accessed on 1 May 2023)) is a website for building PPI networks. Using GeneMANIA, we identified PPI networks of hub biomarkers in this study.

### 2.5. Diagnostic Value of Characteristic Biomarkers and Data Validation in DN

To validate the diagnostic value of the selected hub genes, we built a logistics model and visualized the results with the ggplot2 package. The area under the ROC curve was used to evaluate the biomarkers’ diagnostic value (AUC, which was between 0.5 and 1). The diagnosis is more accurate when the AUC is near 1. Additionally, we also utilized the RNA expression datasets GSE104948 (which included 7 DN samples and 18 control samples) and GSE30529 (which included 10 DN samples and 12 control samples) as validation sets to conduct a controlled reliability study.

### 2.6. RT-qPCR

The SV40-MES-13 mouse mesangial cell line was obtained from BNCC Biological Technology (Beijing, China) and cultured in DMEM medium (Solarbio, Beijing, China) supplemented with 10% fetal bovine serum (FBS; BI) and 1% penicillin/streptomycin. The cells were incubated at 37 °C in a 5% CO2-humidified atmosphere. Control cells were cultured in a normal medium containing 5.5 mM glucose, while the model cells were treated with 40 mM high glucose for 24 h. Total RNA was isolated from the cells using the TRIzol kit (Thermo Fisher Scientific, Waltham, MA, USA) following the manufacturer’s protocol. The isolated RNA was then reverse-transcribed into cDNA using the M-MuLV First Strand cDNA Synthesis Kit (Sangon Biotech, Shanghai, China). Quantitative real-time PCR was performed using the SYBR Premix EX Taq™ II (Tli RNaseH Plus) kit (Takara Bio, Inc., Kusatsu, Japan) and an ABI Stepone plus PCR system (ABI, Oakland, CA, USA). The expression levels of the hub genes were normalized to GAPDH and analyzed using the 2^−ΔΔCt^ method. The primer sequences can be found in Supplementary Table S17.

### 2.7. Evaluation of Immune Cell Infiltration and Correlation Analysis between Diagnostic Markers and Infiltrating Immune Cells

The expression of immune cells plays a crucial role in the development of kidney diseases. In order to estimate the frequency of immunological invasion, the 1000 permutation deconvolution method CIBERSORT [25] converts the expression matrix into different immune cell types. Then, generate a histogram to illustrate the different cell components. A correlation heatmap of different cell components was created to show associations between different subtypes. A box plot was also used to show the differential analysis between immune cells from DN and healthy tissue. A correlation analysis of Spearman’s rank was utilized to examine and illustrate relationships between the detected biomarkers and the quantity of invading immune cells using dot-bar graphs.

### 2.8. Gene Set Enrichment Analysis (GSEA) of Biomarkers

In order to explore the possible roles of the chosen biomarkers in DN, we used the GSEA analysis [26] to explore Human Phenotypic Ontology, GO items, and KEGG pathways. Based on the biomarker expression levels, all samples were split into low-expression groups (50%) and high-expression groups (50%). Reference gene sets included the Molecular Signatures Database datasets c5.go.bp.v7.4.symbols.gmt, c2.cp.kegg.v7.4.symbols.gmt, and c5.hpo.v7.4.symbols.gmt. For GSEA analysis with default settings, *p* < 0.05 was regarded as statistically significant.

### 2.9. Single-Cell Expression Analysis and Subcellular Localization of Biomarkers

On the basis of the HPA database [27,28] (https://www.proteinatlas.org/ (accessed on 1 May 2023)), single-cell data and transcriptional data were utilized to assess the expression of biomarkers in kidney cells. Based on the COMPARTMENTS database (https://compartments.jensenlab.org/ (accessed on 1 May 2023)), we also predicted biomarker protein subcellular localization. This website serves as a prediction tool for proteins’ subcellular locations.

### 2.10. Drug–Protein Interaction and Molecular Docking Analysis of Biomarkers

The Drugbank database (https://go.drugbank.com/ (accessed on 1 May 2023)) was used to identify existing or possibly relevant drug compounds in order to investigate drug–protein interactions. The 3D structures of target proteins and ligands were found using the AlphaFold Protein Structure database (https://alphafold.ebi.ac.uk/ (accessed on 1 May 2023)) and the PubChem database (https://pubchem.ncbi.nlm.nih.gov/ (accessed on 1 May 2023)). Docking simulations and visualizations were performed and presented using PyMOL software 2.3.0 and AutoDock Vina 1.2.0 [29,30].

### 2.11. Statistical Analysis

Statistical analyses were conducted using GraphPad Prism 8.0.2 and R (version 4.2.1). For comparing data between two groups, either Student’s *t*-test or Mann–Whitney U-test was applied, depending on the normality of the data. A significance level of *p* < 0.05 was considered statistically significant. The level of significance was denoted as follows: * *p* < 0.05, ** *p* < 0.01, *** *p* < 0.001, and **** *p* < 0.0001.

## 3. Results

### 3.1. Data Preprocessing

**Figure 2:**
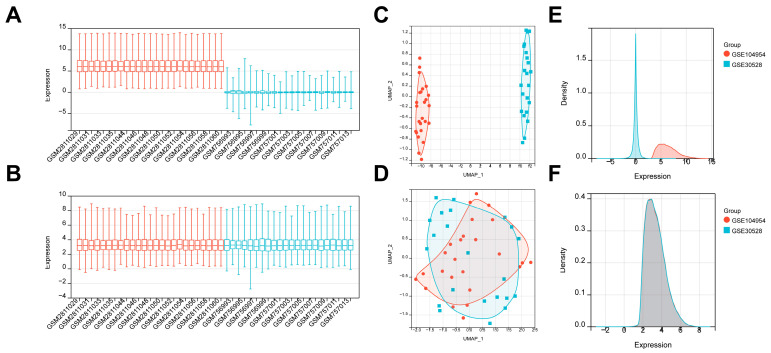
Data preprocessing. (**A**,**B**) Box diagram shows the dataset sample distributions before and after batch removal. (**C**,**D**) UMAP shows the dataset sample distributions before and after batch removal. (**E**,**F**) Density map shows the dataset sample distributions before and after batch removal.

Firstly, to obtain gene expression profiles of DN patients, we downloaded two large-scale clinical datasets, GSE104954 and GSE30528, from the GEO database. We eliminated batch impact from the gene expression matrix after merging the GSE104954 and GSE30528 datasets (Supplementary Tables S2 and S3). The box diagram in Figure 2A,B demonstrated that the datasets’ sample distributions differed significantly before batch impact was eliminated, implying batch variance’s existence. After batch impact removal, the datasets’ sample median distributions tend to be the same. In addition, UMAP findings in Figure 2C,D indicated that the two datasets were independent of one another and did not intersect before batch impact was removed. After batch variance was removed, the sample distributions tended to be similar. Moreover, density curves in Figure 2E,F revealed a substantial variation between the two datasets’ sample distributions prior to batch impact exclusion. After it was removed, the sample distributions between the datasets were almost consistent.

### 3.2. Identification and Function Enrichment of DEGs for DN

**Figure 3:**
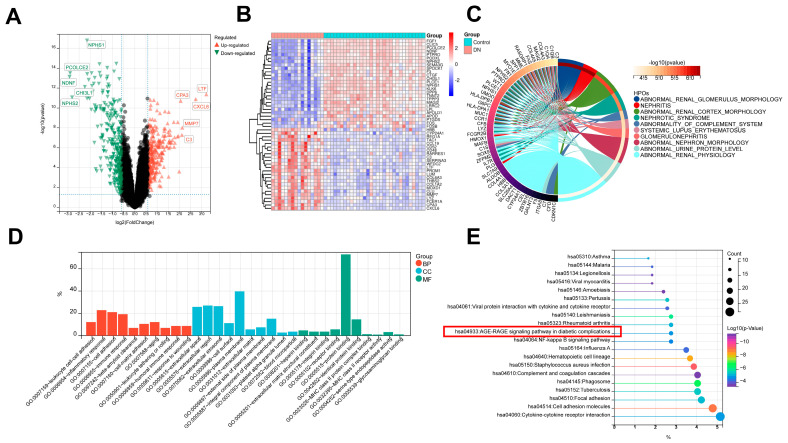
Identification and function enrichment of DEGs for DN. (**A**,**B**) Volcano plot and cluster heatmap show DEGs between the DN and control group. (**C**) Human phenotype ontology analysis for DEGs. (**D**) GO biological processes analysis for DEGs. (**E**) KEGG pathway analysis for DEGs.

We obtained the DEGs from the gene expression matrix after merging the datasets. A total of 559 genes were screened as DEGs under the conditions of *p*-value > 0.05 and |fold-change (FC)| > 1.5, with 270 genes up-regulated and 289 genes down-regulated (Figure 3A,B) (Supplementary Table S4). The biological functions and pathways related to 559 DEGs were then examined using HPO, GO, and KEGG enrichment analyses (Supplementary Tables S5–S7). The top 10 HPO results showed that abnormal renal glomerulus morphology, abnormal renal cortex morphology, nephrotic syndrome, glomerulonephritis, and abnormal urine protein levels were significantly enriched (Figure 3C), which confirmed our data’s dependability. More importantly, the top 10 GO analysis showed that a large number of biological processes related to immune and inflammatory responses were significantly enriched, including inflammatory response, immune response, antigen processing, and presentation of exogenous peptide antigen via MHC class II, positive regulation of T cell activation, positive regulation of ERK1 and ERK2 cascade, and cellular response to interleukin-1 (Figure 3D). In addition, cell adhesion, angiogenesis, and positive regulation of cell migration were also enriched. In terms of the KEGG pathway, complement and coagulation cascades, cytokine-cytokine receptor interaction, the AGE-RAGE signaling pathway in diabetic complications, and the NF-kappa B signaling pathway were significantly enriched (Figure 3E). The findings above clearly imply that inflammation and autoimmunity are crucial components of DN development.

### 3.3. Weighted Gene Co-Expression Network Construction and Identification of Clinically Significant Modules

**Figure 4:**
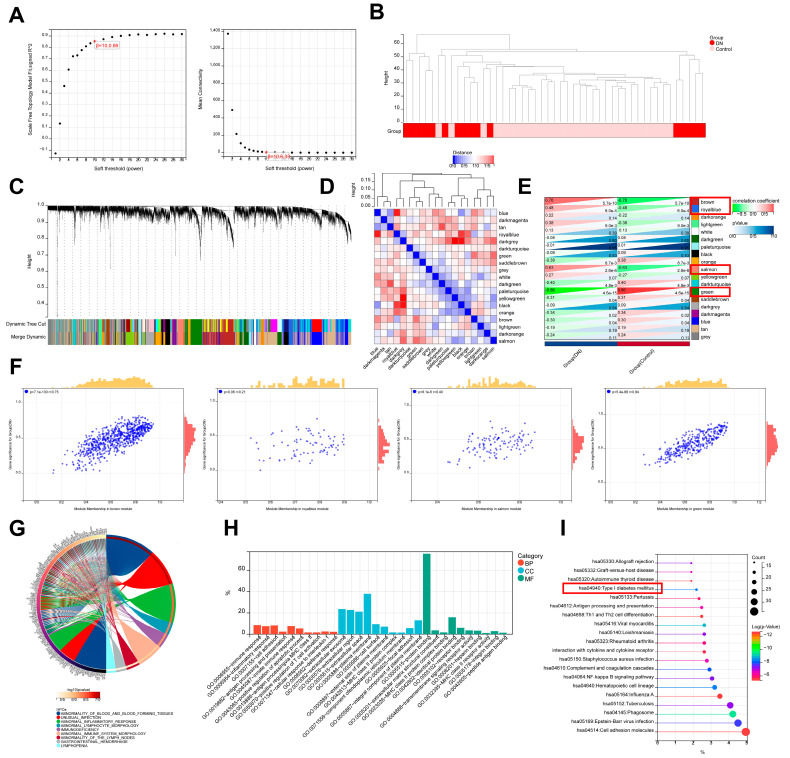
Identification of DN-associated key modules based on WGCNA analysis. (**A**) Scale-free fitting index analysis and mean connectivity of soft threshold power from 1 to 30. (**B**) Clustering dendrogram with tree leaves corresponding to individual samples. (**C**) Clustering dendrogram of all expressed genes based on a dissimilarity measure (1-TOM). (**D**) Correlation heatmap of the module feature vector. (**E**) Correlation heatmap between module eigengene and DN clinical trait. (**F**) Correlation scatter plot between DN gene significance and green module membership. (**G**) Human phenotype ontology analysis for green module genes. (**H**) GO biological processes analysis for green module genes. (**I**) KEGG pathway analysis for green module genes.

In order to determine the critical modules most closely related to DN, WGCNA was carried out using the combined gene expression profile (Supplementary Table S8). Then, when R2 = 0.86 and the average connectivity is high, we set the soft threshold to 10 (Figure 4A,B). After combining strong association modules with a cluster height limit of 0.25, a total of 19 modules were found (Figure 4C). Then, the clustering of module feature vectors was explored, and the results showed the distance between them (Figure 4D). The relationships between modules and clinical symptoms were also investigated. The top 4 results demonstrated the strongest correlation between the “group” attribute (i.e., DN and Control) and the brown module, the royal blue module, the salmon module, and the green module, especially the green module (Figure 4E,F).

To comprehend the biological roles that the green module’s genes perform, we carried out functional enrichment (Supplementary Tables S9–S11). The top 10 HPO results demonstrated that abnormal inflammatory response, abnormal lymphocyte morphology, immunodeficiency, abnormal immune system morphology, abnormality of the lymph nodes, and lymphopenia were significantly enriched (Figure 4G). According to the results of GO and KEGG analysis, DEGs in the green module were related to numerous biological processes and pathways that were linked to infection, inflammation, and autoimmunity. GO enrichment analysis showed that the green module’s genes have immune response, inflammatory response, antigen processing and presentation, innate immune response, antigen processing and presentation of exogenous peptide antigen via MHC class II, positive regulation of T cell activation, and cellular response to interleukin-1 (Figure 4H). In addition, cell adhesion, positive regulation of apoptotic process, and defense response were also enriched, showing their potential pathogenesis in DN. More importantly, KEGG analysis was associated with cell adhesion molecules, Type I diabetes mellitus, complement and coagulation cascades, and NF-kappa B signaling pathway (Figure 4I).

### 3.4. Identification and Function Enrichment of DEARGs for DN

**Figure 5:**
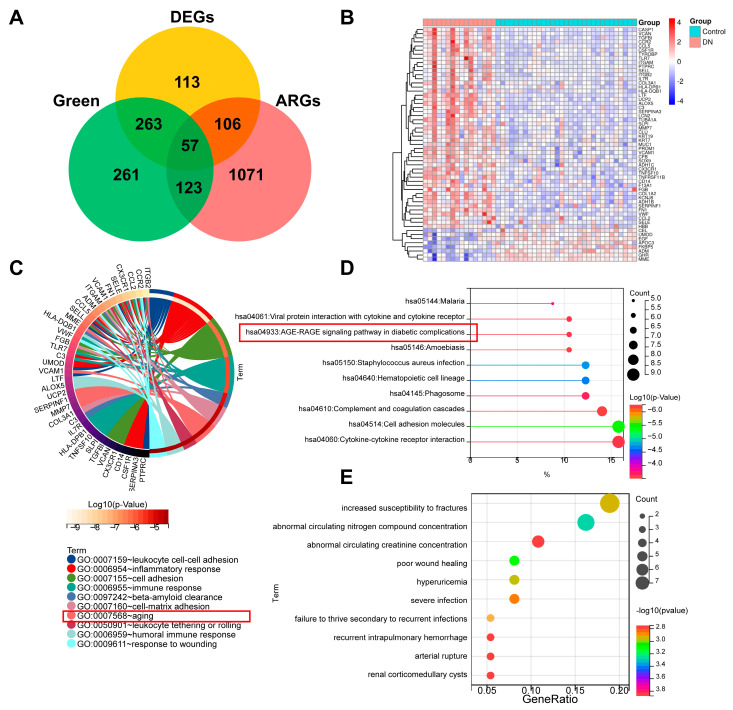
Identification and function enrichment of DEARGs for DN. (**A**) Identification of DEARGs with a Venn diagram. (**B**) Heat map showing differences in DEARGs between the DN and control group. (**C**) Human phenotype ontology analysis for DEARGs. (**D**) GO biological processes analysis for DEARGs. (**E**) KEGG pathway analysis for DEARGs.

Then, the DEGs, the WGCNA green module genes, and aging-related genes were overlapping. We intersected 57 genes (DEARGs) in total (Figure 5A). The heat map displayed the expression features for 57 DEARGs in DN individuals as well as controls (Figure 5B). In addition, a functional analysis was performed on the 57 DEARGs (Supplementary Tables S12–S14). In HPO results, abnormal circulating creatinine concentration, abnormal circulating nitrogen compound concentration, hyperuricemia, severe infection, and renal corticomedullary cysts were enriched (Figure 5C). In BP results, the leukocyte cell-cell adhesion, inflammatory response, immune response, aging, and humoral immune response were enriched (Figure 5D). The KEGG results indicated that complement and coagulation cascades, cell adhesion molecules, AGE-RAGE signaling pathway in diabetic complications, and cytokine-cytokine receptor interaction might participate in the pathogenesis of DN (Figure 5E).

### 3.5. Identification of Hub DEARGs with a Least Absolute Shrinkage and Selection Operator (LASSO) Algorithm

**Figure 6:**
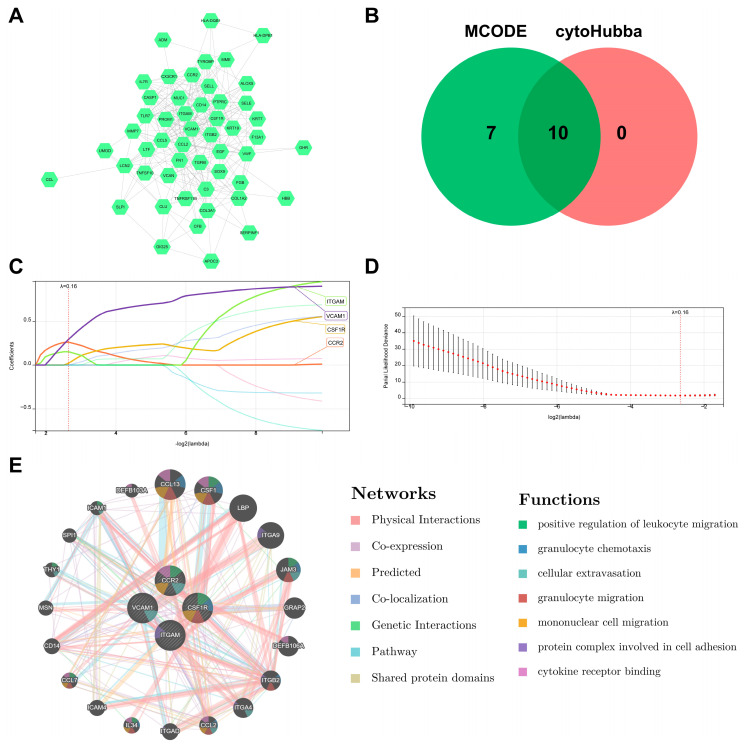
Identification of hub DEARGs of DN. (**A**) PPI network of DEARGs. (**B**) Venn diagram showing the intersections of the DEARGs by cytoHubba and MCODE. (**C**,**D**) Four feature genes with non-zero coefficients were selected by optimal lambda based on the LASSO regression model. (**E**) GeneMANIA database showing DEARGs and their co-expression gene networks.

To further explore DN-associated hub DEARGs and relative mechanisms, we uploaded the aforementioned 57 DEARGs to the STRING website and built a PPI network consisting of 51 nodes and 297 edges (Figure 6A) (Supplementary Table S15). The top 10 genes among the 51 nodes with a high binding degree were identified using the MCODE and MCC calculation algorithms in Cytoscape (Figure 6B). We selected the candidate genes for feature gene screening through LASSO regression. The results of the LASSO regression identified four hub DEARGs (CCR2, VCAM1, CSF1R, and ITGAM) with non-zero regression coefficients and the optimal lambda value of lambda. min = 0.16 (Figure 6C,D).

The GeneMANIA database was then used to study the hub DEARGs co-expression networks and probable roles (Figure 6E) (Supplementary Table S16). We discovered a complex PPI network with 0.60% protein domain, 1.88% pathway, 2.87% genetic relationships, 3.64% co-localization, 5.37% predicted interactions, 8.01% co-expression, and 77.64% physical interactions. According to a function study, they were mostly linked to various immunological and inflammatory processes, including positive regulation of leukocyte migration, granulocyte chemotaxis, cellular extravasation, granulocyte migration, mononuclear cell migration, a protein complex involved in cell adhesion, and cytokine receptor binding, indicating their critical involvement in the etiology of DN.

### 3.6. RT-qPCR and Datasets Validation and Diagnostic Value of Hub DEARGs for DN

**Figure 7:**
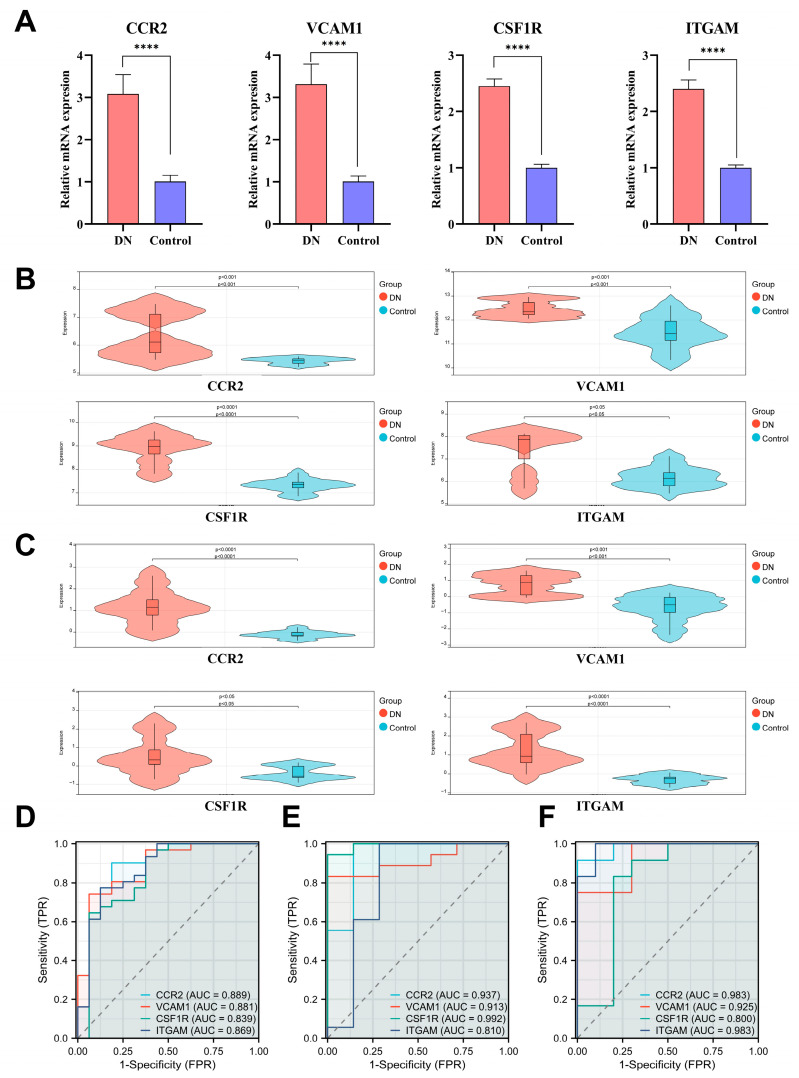
RT-qPCR and datasets validation, and diagnostic value of hub DEARGs for DN. (**A**) RT-qPCR validation of hub DEARGs. (**B**) Dataset validation of hub DEARGs by GSE104948. (**C**) Dataset validation of hub DEARGs by GSE30529. (**D**) ROC curves estimate the diagnostic values of DEARGs in merged datasets. (**E**) ROC curves estimate the diagnostic values of DEARGs in GSE104948. (**F**) ROC curves estimate the diagnostic values of DEARGs in GSE30529. (Note: **** *p* < 0.0001).

In order to confirm the expression of CCR2, VCAM1, CSF1R, and ITGAM in DN, we created an in vitro high glucose-induced human mesangial cell model. We discovered that, compared to control subjects, these proteins were all substantially expressed in the cells of the model group, revealing the accuracy of our bioinformatics predictions (Figure 7A) (Supplementary Table S17). Further analysis was done on the other two new DN-related datasets, GSE104948 and GSE30529 (Figure 7B,C). Verification revealed that hub biomarker expressions were all higher in DN groups than in control groups (Supplementary Table S18). The results fully validated the presumption that CCR2, VCAM1, CSF1R, and ITGAM may serve as DN diagnostic biomarkers.

Next, we used ROC curves to investigate the association between hub DEARG expressions and DN patient prognosis in order to evaluate the potential predictive usefulness of four hub markers in DN (Supplementary Table S19). High diagnostic specificity and sensitivity for DN were regarded as having an AUC greater than 0.800. According to Figure 7D, CCR2 is 0.889 (95% CI: 0.775–1.000), VCAM1 is 0.881 (95% CI: 0.777–0.986), CSF1R is 0.839 (95% CI: 0.700–0.978), and ITGAM is 0.869 (95% CI: 0.749–0.989). Moreover, the AUC values of two new DN-related datasets also showed excellent diagnostic utility (Figure 7E,F). The findings demonstrated the high DN diagnostic value of CCR2, VCAM1, CSF1R, and ITGAM.

### 3.7. Immune Cell Infiltration Analysis

**Figure 8:**
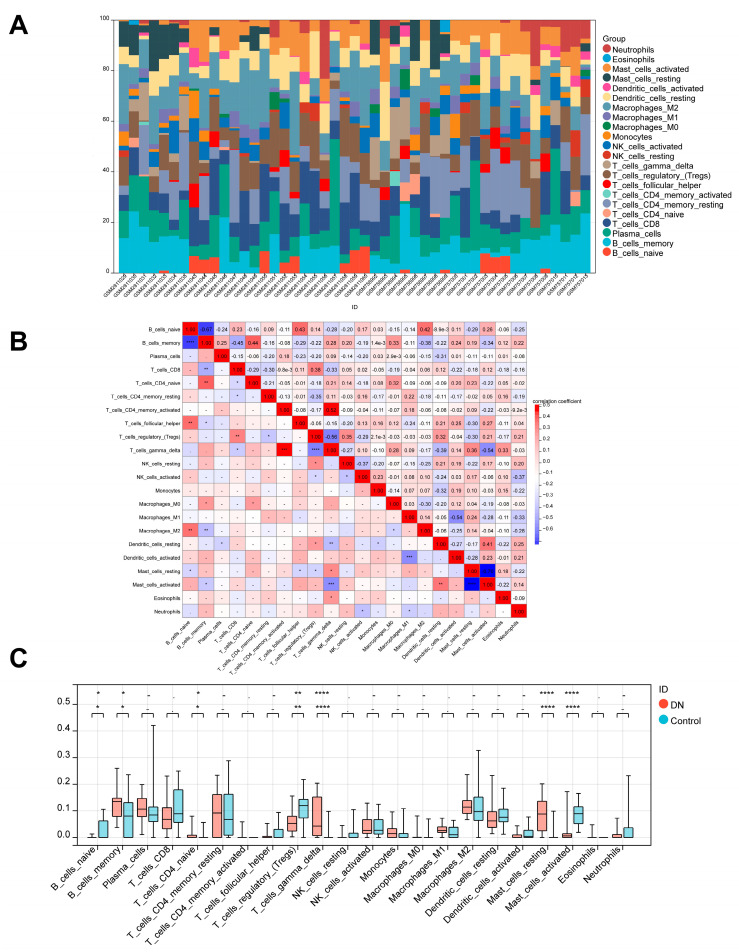
Immune infiltration analysis of DN. (**A**) Each sample’s proportion of different immune cells. (**B**) Correlation between different immune cells. (**C**) Expression abundance of different immune cells in DN and control. (Note: * *p* < 0.05, ** *p* < 0.01, *** *p* < 0.001 and **** *p* < 0.0001).

To analyze immunological patterns in DN and normal tissues, we used CIBERSORT to compute the proportion of 22 immune cells in each sample through the matrix of gene expression (Supplementary Table S20). Each sample’s 22 different categories of immune cells were represented by a histogram (Figure 8A). Each histogram’s colors showed the immune cell percentages, with a sum of 1 for each sample. The findings showed that the most prevalently infiltrated immune cells in all 47 samples were resting dendritic cells (47), plasma cells (46), regulatory T cells (46), and M2 macrophages (46). Eosinophils (2), CD4 memory resting T cells (2), and CD4 naive T cells (7) were infiltrating less, though. In the subsequent study, the correlation between 22 categories of immuno-infiltrated cells in two groups was investigated (Figure 8B). Delta gamma T cells were strongly positively correlated with activated CD4 memory T cells and negatively correlated with regulatory T cells and activated mast cells, according to the correlation heat map of different immune cells. Violin plots of the differential in immune cell infiltration revealed that compared to the normal control sample, memory B cells, naive CD4 T cells, delta gamma T cells, and resting mast cells infiltrated more, whereas naive B cells, regulatory T cells, and activated mast cells infiltrated less (Figure 8C).

### 3.8. Correlation between Hub DEARGs and Immune Cells

**Figure 9:**
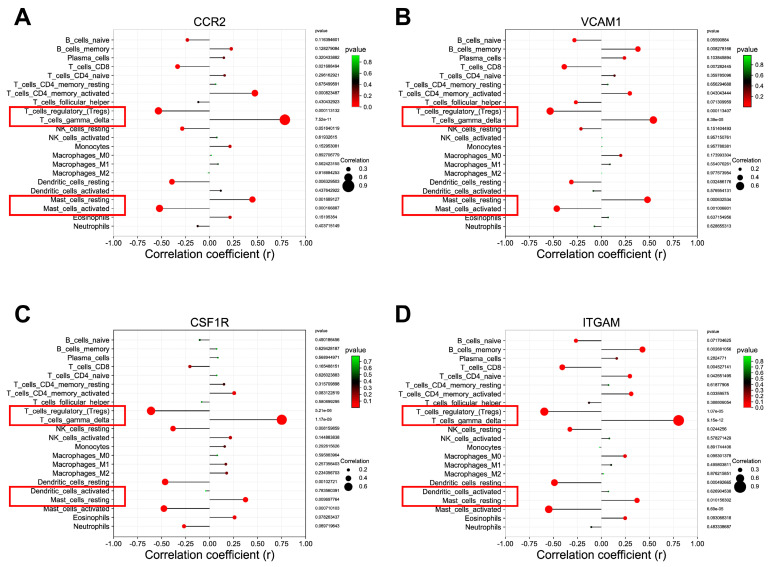
Correlation between the hub DEARGs and different immune cells. (**A**) CCR2; (**B**) VCAM1; (**C**) CSF1R; (**D**) ITGAM.

We next investigated the relationship between hub DEARG expression and immune cell abundance (Supplementary Table S21). The results of Pearson’s correlation showed that a total of four types of immune cells were associated with all four DEARGs. As shown in Figure 9A–D, regulatory T cells and active mast cells were statistically negatively correlated with CCR2, VCAM1, CSF1R, and ITGAM, but delta gamma T cells and resting mast cells were positively correlated with them, suggesting they may play crucial roles in DN formation.

### 3.9. GSEA of Hub DEARGs

**Figure 10:**
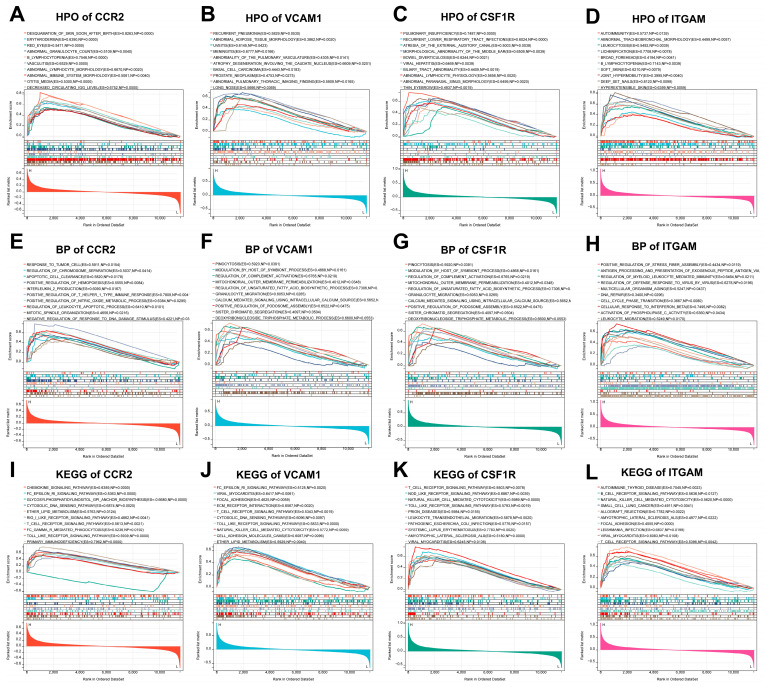
GSEA of hub DEARGs. (**A**–**D**) Human phenotype ontology analysis for (**A**) CCR2, (**B**) VCAM1, (**C**) CSF1R, and (**D**) ITGAM. (**E**–**H**) GO biological processes analysis for (**E**) CCR2, (**F**) VCAM1, (**G**) CSF1R, and (**H**) ITGAM. (**I**–**L**) KEGG pathway analysis for (**I**) CCR2, (**J**) VCAM1, (**K**) CSF1R, and (**L**) ITGAM.

Furthermore, we investigated the precise signaling pathways and the probable biological processes of the hub DEARGs that regulated DN development (Supplementary Tables S22–S25). The top 10 GSEA HPO results revealed that CCR2 was mostly relevant for abnormalities in a variety of immune cells, including abnormal granulocyte count, B lymphocytopenia, vasculitis, abnormal lymphocyte morphology, and abnormal immune system morphology (Figure 10A). The main enriched items for VCAM1 were recurrent pneumonia, meningitis, and basal cell carcinoma (Figure 10B). The main enriched items for CSF1R were recurrent lower respiratory tract infections, viral hepatitis, and abnormal lymphocyte physiology (Figure 10C). As for ITGAM, the main enriched items were autoimmunity, leukocytosis, and B lymphocytopenia (Figure 10D).

What is more, the top 10 GO BP results revealed that CCR2 regulated a variety of immunological responses, including regulation of chromosome separation, apoptotic cell clearance, interleukin-2 production, positive regulation of T helper 1 type immune response, and regulation of leukocyte apoptotic process (Figure 10E). The main enriched terms for VCAM1 were regulation of complement activation, mitochondrial outer membrane permeabilization, regulation of unsaturated fatty acid synthetic processes, granulocyte migration, and calcium-mediated signaling using intracellular calcium (Figure 10F). The main enriched terms for CSF1R expression were leukocyte differentiation, lymphocyte activation, leukocyte migration, apoptotic cell clearance, and regulation of lymphocyte activation (Figure 10G). As for ITGAM, the main enriched terms were antigen processing and presentation of exogenous peptide antigen via MHC class I, regulation of myeloid leukocyte-mediated immunity, regulation of defense response to virus by virus, and multicellular organism aging (Figure 10H).

Meanwhile, KEGG gene sets found that CCR2 was primarily enriched in the chemokine signaling pathway, the T cell receptor signaling pathway, Fc gamma R mediated phagocytosis, the Toll-like receptor signaling pathway, and primary immunodeficiency (Figure 10I). The main enriched pathways for VCAM1 were the Fc epsilon ri signaling pathway, ECM receptor interaction, T cell receptor signaling pathway, Toll-like receptor signaling pathway, and natural killer cell-mediated cytotoxicity (Figure 10J). The main enriched pathways for CSF1R were the T cell receptor signaling pathway, the Nod-like receptor signaling pathway, natural killer cell-mediated cytotoxicity, the Toll-like receptor signaling pathway, and leukocyte transendothelial migration (Figure 10K). As for ITGAM, the main enriched pathways were the B cell receptor signaling pathway, natural killer cell-mediated cytotoxicity, allograft rejection, focal adhesion, and the T cell receptor signaling pathway (Figure 10L). The above results suggest that all of these hub DEARGs might play essential roles in the regulation of immunity and inflammation in DN.

### 3.10. Single Cell Analysis and Subcellular Localization of Hub DEARGs

**Figure 11:**
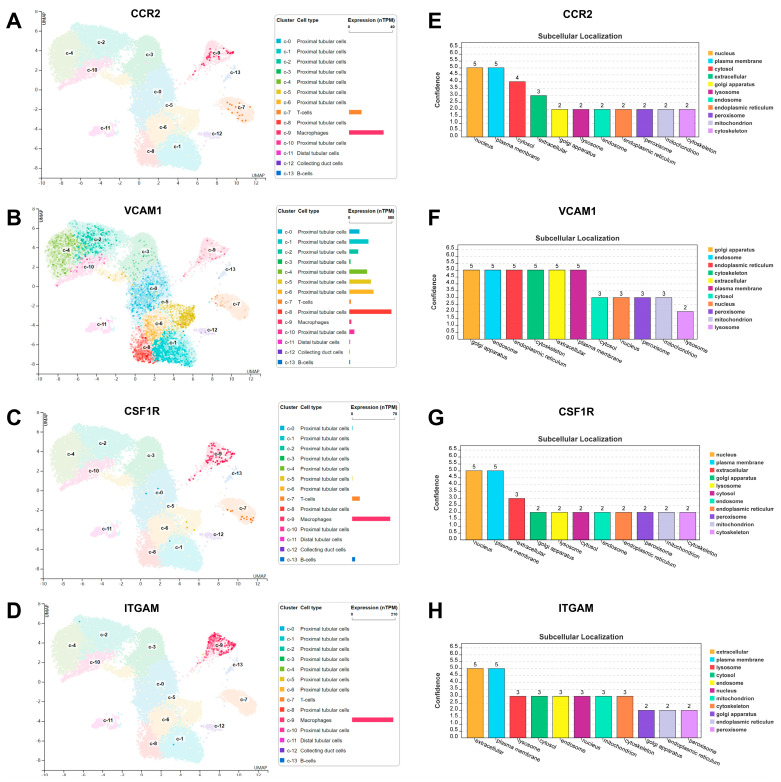
Single-cell expression analysis and subcellular localization of hub DEARGs. (**A**–**D**) Single-cell expression analysis for (**A**) CCR2, (**B**) VCAM1, (**C**) CSF1R, and (**D**) ITGAM. (**E**–**H**) Subcellular localization of (**E**) CCR2, (**F**) VCAM1, (**G**) CSF1R, and (**H**) ITGAM.

To more precisely delineate the expression of hub DEARGs in human kidney tissues, we interrogated a scRNA-seq based on the HPA database to identify the cell populations expressing in DN. Clustering identified 15 kidney cell subpopulations, as shown in the UMAP plot. The outcomes further revealed the major expression of CCR2 in macrophages and T cells (Figure 11A), VCAM1 in proximal tubular cells (Figure 11B), CSF1R in macrophages, T cells, and B cells (Figure 11C), and ITGAM in macrophages (Figure 11D). Proteins have different biological functions depending on where they are in the cell. Based on the COMPARTMENTS database (Supplementary Table S26), we further predicted the protein subcellular localization of hub DEARGs. CCR2 is primarily distributed in the nucleus and plasma membrane (Figure 11E), VCAM1 is primarily distributed in the Golgi apparatus, endosome, endoplasmic reticulum, cytoskeleton, extracellular, and plasma membrane (Figure 11F), CSF1R is primarily distributed in the nucleus and plasma membrane (Figure 11G), and ITGAM is primarily distributed in extracellular and plasma membrane (Figure 11H).

### 3.11. Drug–Gene Interaction and Molecular Docking Analysis of Hub DEARGs

**Figure 12:**
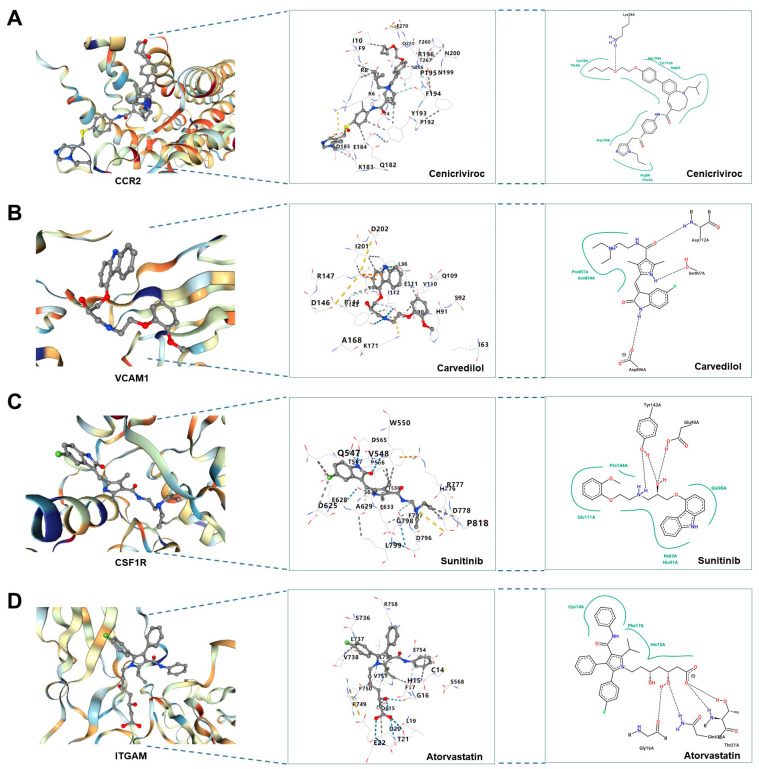
Drug–protein interactions based on molecular docking. (**A**) Molecular docking of CCR2 and cenicriviroc. (**B**) Molecular docking of VCAM1 and carvedilol. (**C**) Molecular docking of CSF1R and sunitinib. (**D**) Molecular docking of ITGAM and atorvastatin.

Developing possible therapeutic medicines that target CCR2, VCAM1, CSF1R, and ITGAM offers a unique therapy strategy. Four small molecular medicines, including cenicriviroc, carvedilol, sunitinib, and atorvastatin, were ultimately obtained based on the GeneCards database (Table 1). The potential for binding was then assessed by docking the aforementioned four bioactive chemical ligands with the proteins CCR2, VCAM1, CSF1R, and ITGAM. The docking 3D and 2D models of the proteins CCR2, VCAM1, CSF1R, and ITGAM, as well as four small-molecule medications with the firmest binding, were shown in Figure 12A–D, demonstrating their ability to lessen or even reverse the development of DN (Supplementary Table S27).

## 4. Discussion

The aging of the kidneys is a complicated process that interacts with a variety of disorders, particularly those that are more common in the elderly. Glomerular filtration rate (GFR) decline, a physiological feature of DN, is a manifestation of kidney aging [31,32]. Beyond age 35, the GFR declines by roughly 5–10% per decade, while those between the ages of 18 and 29 had 48% more intact nephrons than those between the ages of 70 and 75 [33,34]. The increase in senescent cells leads to two main effects. First, as one might anticipate, the senescence of cells may result in a lack of self-repair and regeneration potential due to persistent cell cycle arrest [35,36,37,38]. This might cause other progenitor or stem cells, in addition to renal cells, to run out. According to a study, people with chronic renal disease have 30–50% fewer endothelial progenitor cells than healthy subjects [39]. In kidneys with DN, there is a small reservoir, a diminished population, and a low rate of stem cell renewal [40,41], all of which would inevitably hasten the disease’s course. Second, the aging process also affects the immune system, including immune-cell function and biomolecules that act as effectors, which results in immunosenescence, immunoactivation, and inflammatory processes [42,43]. Cytokines that promote inflammation and matrix synthesis, such as IL-6 and TGF-β, can be produced by senescent cells. These SASP-associated compounds may have a paracrine and autocrine role in stem and kidney cell renewal, as well as persistent inflammation and fibrosis [44,45]. In summary, cellular senescence engages in various pathogenic processes collectively to promote the development of DN.

In this work, we screened 559 DEGs and discovered 289 down-regulated genes and 270 up-regulated genes. Further GO enrichment analysis revealed that a large number of biological processes associated with immunological and inflammatory responses (inflammatory response, immune response, positive regulation of T cell activation, and cellular response to interleukin-1) are considerably enriched, whereas a KEGG enrichment study revealed some correlation with complement and coagulation cascades, cytokine-cytokine receptor interaction, the AGE-RAGE signaling pathway in diabetic complications, and the NF-kB signaling pathway. Also, the results above are further supported by Human Phenotype Ontology analysis. The primary enriched terms were abnormal renal glomerulus morphology, abnormal renal cortex morphology, nephrotic syndrome, glomerulonephritis, and abnormal urine protein level. As a result, it is possible that the DEGs have a role in DN pathogenesis.

Then, based on WGCNA analysis, we discovered 19 DN-related modules. Many biological functions and pathways associated with inflammation and the immune system have been revealed to involve DEGs in the green module. We got a total of 57 DEARGs by overlapping the DEGs, the green module genes, and the ARGs. To further identify DN-related hub DEARGs, we estimated the expression of the aforementioned 57 DEARGs using MCODE, MCC, and LASSO methods and then identified four DEARGs (CCR2, VCAM1, CSF1R, and ITGAM). Fewer of them have been reported in the development of DN, but many of them have been linked to immunological and inflammatory responses in other disorders.

C-C chemokine receptor type 2 (CCR2) modulates the immune response by causing the migration of macrophages and monocytes to areas of inflammation [46,47]. In fact, it has been demonstrated that CCR2 is engaged in the DN process. According to research, macrophages play a direct role in the renal injury of diabetic nephropathy, and genetic CCR2 deletion provides kidney protection in renal injury [48]. The 90-kDa glycoprotein known as Vascular Cell Adhesion Molecule 1 (VCAM-1) is mostly expressed in endothelial cells. The first identification of VCAM-1 as an endothelial cell surface glycoprotein occurred in 1989 [49,50]. Inflammatory cytokines, including TNF, ROS, oxidized LDL, elevated blood sugar levels, toll-like receptor agonists, and shear stress, all stimulate the production of VCAM-1 [51]. A class of tyrosine/serine kinases known as colony-stimulating factor 1 receptor (CSF1R) are primarily in charge of controlling the proliferation and differentiation of microglia and macrophages [52]. In both human and rat RA models, CSF1R blockade lowers inflammation [53]. CSF1R is bound by CSF, which increases cell survival and proliferation [54]. In RA, synovial endothelial cells generate CSF [55] and IL-1β and TNF [56] in vitro. Integrin Subunit Alpha M (ITGAM) encodes the CD11b-subunit of the Mac1 or CD11b/CD18 integrin, which has been repeatedly linked to susceptibility to systemic lupus erythematosus (SLE) [57]. In diabetic nephropathy, ITGAM may contribute to kidney injury by increasing macrophage recruitment in the kidneys and causing histological abnormalities in the glomeruli [58].

For a comprehensive understanding of the dysregulated immune cells in DN, an immune infiltration investigation was conducted. We discovered that DN tissue had higher levels of resting mast cells and delta gamma T cells but lower levels of regulatory T cells and activated mast cells. Moreover, our investigation demonstrated that several main immune cells were statistically correlated to all four DEARGs (CCR2, VCAM1, CSF1R, and ITGAM). For instance, delta gamma T cells and resting mast cells showed a positive correlation with all hub genes, but regulatory T cells and activated mast cells showed a negative correlation with them. They might, therefore, be extremely important in the immunomodulation of DN and be linked to the malfunctioning of inflammatory cells in DN. The immunological response of effector T cells, B cells, and innate immune cells is suppressed by CD4+ T cells known as Tregs (regulatory T cells). Tregs limit inflammatory immunity in numerous ways, including through the renal and systemic systems [59]. Current research indicates that kidney disease may cause a decline in the proportion of Tregs and an impairment of their regulatory capabilities [60]. Delta gamma T cells are the primary warriors of both the innate and adaptive immune systems, making up an average of 3.7% of CD3+ T cells in peripheral blood [61,62]. The fact that delta gamma T cells are classified as T lymphocytes have TCR rearrangement, have the capacity to create immunological memory, and can lyse target cells all point to them being an important component of the adaptive immune system [63]. Studies have shown that T lymphocytes control renal operational processes. Mast cells are mononuclear, non-dividing cells that are a component of the innate immune system [64]. The two phenotypes of mast cells that are typically distinguished are those that secrete tryptase and chymase and those that exclusively secrete tryptase. Tryptase-secreting mast cells may perform many functions in the immunological response, whereas chymase-secreting mast cells may also participate in revascularization and tissue repair [65,66]. The release of mediators by mast cells during inflammation may result in the loss of kidney structure, and mast cells have also been recognized as significant effector cells in renal inflammation [67]. Despite this, there are not many researches that investigate how hub DEARGs relate to resting memory CD4 T cells, gamma delta T cells, and mast cells in the DN, which could be a fascinating discovery.

Following that, we looked into the particular signal pathways that the four hub DEARGs enriched and investigated how the hub DEARGs might affect the development of DN. Four DEARGs were implicated in immune cell abnormalities, and GSEA analysis revealed that they controlled a great deal of immune system responses and inflammatory pathways, including apoptosis, interleukin-2 production, complement activation, Nod-like receptor signal pathways, and Toll-like receptor signal pathways, indicating that hub DEARGs may be a potential biomarker for DN diagnosis and prognosis. Apoptosis, a sort of active, programmed cell death, maintains the stability of the body’s environment [68]. Apoptosis and proliferation of cells are directly controlled by genes, ensuring the equilibrium state of the body’s cells [69]. It has been discovered that apoptosis has a significant impact on glomerular remodeling and regulates glomerular cell regression during CGN recovery [70,71]. Interleukin 2 (IL-2) is a multipotent cytokine with a 15.5 kDa four-helix bundle that plays a crucial role in immune control. Antigen-stimulated CD4+ T cells are the predominant producers, but NK T cells, CD8+ T cells, mast cells, and dendritic cells can also generate it [72,73,74,75,76]. Long-term IL-2 treatment decreased the activity and proliferation of intrarenal conventional CD4+ T cells, which was accompanied by a clinical and histological improvement of lupus nephritis, according to research, while short-term IL-2 treatment increased the intrarenal Treg population in mice with active lupus nephritis [77]. Moreover, IL-2 often protects against caspase-8-mediated apoptotic injury, making it a potentially new and practical method to avoid tubular injury in autoimmune kidney diseases [78]. Complement and coagulation cascades perform significant functions in inflammatory-related events and the immune system’s protection and regulation [79]. Coagulation, complement, the fibrinolysis system, and platelets all work together to build a tight network in the blood circulation. Systemic lupus erythematosus, C3 glomerulonephritis, and ischemia-reperfusion damage are just a few examples of illnesses that can advance clinically as a result of dysregulation of any cascade system [80]. A family of pattern recognition receptors (PRRs) known as the NOD-like receptor (NLR) family of proteins is known to mediate the early innate immune response to cellular injury and stress. Its activation occurs not only in immune cells but also in resident cells, including endothelial cells and podocytes in the glomeruli [81,82]. Inflammation and other cellular damage are the results of NLRP3 inflammasome activation, which has been linked to ESRD and glomerular injury in studies [83]. Similar to this, the toll-like receptor family (TLRs) plays a crucial manipulative function in the innate immune system. According to a current study, the transduction of TLR signals affects how the kidney responds to various external and internal stimuli by triggering its inflammatory response [84]. Besides, TLRs also play new roles in addition to their well-known ones in host defense, such as regulating body homeostasis and healing wounds [85].

Moreover, we explored cell subpopulations and the subcellular localization of hub DEARGs in the kidney. The results identified 15 kidney cell subpopulations and predicted various organelles, such as the Golgi apparatus, endosome, endoplasmic reticulum, cytoskeleton, extracellular, and plasma membrane. However, hub DEARGs are mostly expressed in macrophages as well as on the cell membrane in the kidney. Lastly, we discovered four prospective therapeutic medicines that target hub DEARGs, suggesting a potential therapeutic strategy for DN. According to molecular docking, precise molecule binding strengthens the reliability of this association.

Our investigation was subject to certain limitations. Specifically, we quantified gene expression levels exclusively in high glucose-induced mesangial cells rather than in actual tissue samples. Although this approach provided a representative model, it may not fully capture the intricacies of the in vivo setting. To address this, we intend to conduct future experiments utilizing clinical samples, thereby offering a more direct validation of our findings. By doing so, we aim to enhance the robustness and clinical relevance of our results.

## 5. Conclusions

In conclusion, four potential aging-related genes (CCR2, VCAM1, CSF1R, and ITGAM) associated with DN were identified in this study using bioinformatic analysis and machine learning methods. By controlling senescence, these genes may influence the development and prognosis of DN, and they may also aid in the development of future therapeutic approaches.

## Figures and Tables

**Table 1:** The details of the candidate small molecular drugs targeting hub DEARGs.

DRUGBANK ID	NAME	TYPE	Chemical Formula	DRUG GROUP	ACTIONS
DB11758	Cenicriviroc	Small Molecule	C41H52N4O4S	investigational	inhibitor
DB01136	Carvedilol	Small Molecule	C24H26N2O4	approved, investigational	inhibitor
DB01268	Sunitinib	Small Molecule	C22H27FN4O2	approved, investigational	inhibitor
DB01076	Atorvastatin	Small Molecule	C33H35FN2O5	approved	inhibitor

## Data Availability

The data used and analyzed to support the findings of this study are available from the corresponding author upon request.

## Supplementary Materials

The following supporting information can be downloaded at https://www.mdpi.com/article/10.3390/biomedicines11092454/s1, Table S1: Aging-related genes from GeneCards. Table S2: The merged datasets before batch removal. Table S3: The merged datasets after batch removal. Table S4: Differentially expressed mRNAs in DN. Table S5: The results of Human phenotype ontology enrichment analyses. Table S6: The results of GO enrichment analyses. Table S7: The results of KEGG enrichment analyses. Table S8: The results of differential mRNAs in 19 modules based on WGCNA analysis. Table S9: The results of Human phenotype ontology enrichment analyses. Table S10: The results of GO enrichment analyses. Table S11: The results of KEGG enrichment analyses. Table S12: The results of Human phenotype ontology enrichment analyses. Table S13: The results of GO enrichment analyses. Table S14: The results of KEGG enrichment analyses. Table S15: PPI interactions based on STRING database. Table S16: The results of GeneMANIA database. Table S17: The results of RT-qPCR. Table S18: Hub DEARGs expression data of GSE104948 and GSE30529. Table S19: Hub DEARGs expression data for ROC analysis. Table S20: Calculation of 22 types of immune cells in each sample based on CIBERSORT. Table S21: Correlation analysis between hub genes and immune cells. Table S22: The results of functional enrichment analyses for CCR2. Table S23: The results of functional enrichment analyses for VCAM1. Table S24: The results of functional enrichment analyses for CSF1R. Table S25: The results of functional enrichment analyses for ITGAM. Table S26: The subcellular localization of hub DEARGs. Table S27: The details of molecular docking for hub DEARGs.
